# Recognizing noncommunicable diseases as a global health security threat

**DOI:** 10.2471/BLT.17.205732

**Published:** 2018-10-01

**Authors:** Amrita Saha, George Alleyne

**Affiliations:** a Department of International Health, Johns Hopkins Bloomberg School of Public Health, Baltimore, United States of America (USA).; b Pan American Health Organization, 525 Twenty Third Street NW, Washington, DC, USA.

Protecting health against potential risks such as epidemiological risks that determine disease outbreaks and pandemics, safety risks associated with poor quality of care and financial risks derived from paying for care, will ensure health security.^1^ However, health security can have different meanings. Health security can be understood as securing health at the individual, national and global levels, but may also be understood as the effect of health on security. The latter is a traditional approach that focuses mainly on national security and the protection of sovereignty, borders, people, and private interests and property.^2^ The discrepancy in meanings has caused confusion and mistrust between and among Member States.^3^ In this paper, we discuss securing health from noncommunicable disease at the individual, national and global levels.

A recent *Lancet* editorial noted that noncommunicable diseases are not garnering the attention they deserve and suggested that such diseases should be considered as a global health security issue.^4^ A *Lancet* editorial discussing the 2007 World Health Report called for leadership from the World Health Organization (WHO) to ensure that global health security is achieved.^5^

The impact of noncommunicable diseases on public health is well known. In 2010, 34.5 million out of a total of 52.84 million deaths were attributed to noncommunicable diseases, and most of these occured in low- and middle-income countries.^6^ In 2011, the General Assembly adopted a resolution on the prevention and control of noncommunicable diseases. This political declaration was largely an acknowledgement of the burden of noncommunicable diseases and the role of governments and other stakeholders in preventing and managing this burden. Noncommunicable diseases have also been included in the sustainable development goals with a specific target.^7^

Despite many efforts by WHO and the international community, however, funding for the prevention and control of noncommunicable diseases has lagged. Of the total 37.6 billion United States dollars (US$) in development assistance for health for 2016, 29.4% was allocated to maternal, newborn and child health, 25.4% to human immunodeficiency virus (HIV), 6.6% to malaria, 4% to tuberculosis and 1.7% to noncommunicable diseases.^8^ The scarce funding for noncommunicable diseases is a possible indicator of their low priority on the global health agenda. Here we argue that this situation is in part due to the failure to recognize noncommunicable diseases as a global health security threat.

For example, in contrast with noncommunicable diseases, HIV, an epidemic of global significance, has attracted considerable funding. The security concerns associated with HIV were so pressing that the issue reached the United Nations Security Council. HIV is considered a national security threat because of the impact on strategically important population groups, such as soldiers and peacekeepers and because of its potential to destabilize states.

Noncommunicable diseases can affect personal security in many ways: they are chronic conditions and therefore have a long-lasting impact on health and on the perception of one’s personal security and well-being. Evidence suggests that noncommunicable diseases contribute to personal poverty, because of their chronic nature, their impact on productivity and their direct and indirect costs. However, it is the scale of the premature mortality due to noncommunicable diseases, with its impact on individuals and families, that mainly threatens personal security. The WHO *Global status report on noncommunicable diseases 2014* showed that in 2012, 42% of all deaths caused by noncommunicable diseases occurred before the age of 70 years and 82% were in low- and middle-income countries.^9^ Noncommunicable diseases clearly have an impact on individuals; however, they also represent an economic burden to governments, and therefore are a health security challenge at the national level.

The global dimension of noncommunicable diseases as a health security issue refers to the health of all the people and efforts to reduce health inequity. The Lancet Commission on Global Health 2035 foresees that the threat of pandemics, antimicrobial resistance and noncommunicable diseases will represent the greatest threats to global public health in the future.^10^ Antimicrobial resistance and pandemics have a high priority status in the global agenda and their threat to global health security is largely unquestioned. The West African Ebola outbreak prompted the creation of a global health security agenda. Interestingly, the initiative did not come from the public health community, but from the highest political levels. In 2014, the United States of America, with initially 40 partners from around the world, launched the global health security agenda with the aim to prevent, detect and respond to infectious disease threats globally.^11^ The urge for a rapid response to infectious diseases is not surprising, as fear of contagion is strong; noncommunicable diseases do not pose such a threat and are therefore not perceived as threatening.^12^

We propose that the magnitude of the epidemic of noncommunicable diseases, their increasing prevalence, global costs, potential to overwhelm the response capacity of low-income countries and their contribution to the inequality of health, make noncommunicable diseases a global health security threat. For example, the increased burden of noncommunicable diseases on low-income countries that have inadequate health systems might increase global inequality and instability.

The attention given to a public health issue mainly depends on how the issue is framed.^13^ For noncommunicable diseases to be understood as a global health security issue, perhaps they need to be framed not only in terms of data on morbidity and mortality, or on their economic costs. Leadership to advocate for noncommunicable diseases as a global health security issue is a whole-of-society responsibility, but those who can push for this are intergovernmental organizations such as WHO, and increasingly nongovernmental organizations with global reach such as the Noncommunicable diseases Alliance.

We support the proposal that we should avoid the reductionist approach that limits health security to the control of outbreaks.^14^ It is time that noncommunicable diseases is recognized as a threat to global health security.

## References

[1] Frenk J. Strengthening health systems to promote security. Lancet. 2009 6 27;373(9682):2181–2. 10.1016/S0140-6736(09)60002-7 19150131

[2] Holsti KJ. The diplomacy of security. In: Cooper AE, Heine J, Thakur R, editors. The Oxford Handbook of Diplomacy. Oxford: Oxford University Press; 2013 pp. 377–92.

[3] Aldis W. Health security as a public health concept: a critical analysis. Health Policy Plan. 2008 11;23(6):369–75. 10.1093/heapol/czn030 18689437

[4] Horton R. Offline: NCDs-why are we failing? Lancet. 2017 7 22;390(10092):346. 10.1016/S0140-6736(17)31919-0 28745593

[5] WHO fails to address health security. Lancet. 2007 9 1;370(9589):714. 10.1016/S0140-6736(07)61350-6 17765501

[6] Lozano R, Naghavi M, Foreman K, Lim S, Shibuya K, Aboyans V, et al. Global and regional mortality from 235 causes of death for 20 age groups in 1990 and 2010: a systematic analysis for the Global Burden of Disease Study 2010. Lancet. 2012 12 15;380(9859):2095–128. 10.1016/S0140-6736(12)61728-0 23245604 PMC10790329

[7] Indicators SDG. metadata repository. New York: United Nations; 2018. Available from: https://unstats.un.org/sdgs/metadata/ [cited 2018 Feb 21].

[8] Dieleman JL, Schneider MT, Haakenstad A, Singh L, Sadat N, Birger M, et al. Development assistance for health: past trends, associations, and the future of international financial flows for health. Lancet. 2016 6 18;387(10037):2536–44. 10.1016/S0140-6736(16)30168-4 27086170

[9] World Health Organization global status report on noncommunicable diseases 2014. Geneva: World Health Organization; 2014. Available from: http://www.who.int/nmh/publications/ncd-status-report-2014/en/ [cited 2018 Feb 21].

[10] Jamison DT, Summers LH, Alleyne G, Arrow KJ, Berkley S, Binagwaho A, et al. Global health 2035: a world converging within a generation. Lancet. 2013 12 7;382(9908):1898–955. 10.1016/S0140-6736(13)62105-4 24309475

[11] Frieden TR, Damon I, Bell BP, Kenyon T, Nichol S. Ebola 2014 - New challenges, new global response and responsibility. N Engl J Med. 2014;371(13):1177–80. 10.1056/NEJMp1409903 25140858

[12] Alleyne G, Basu S, Stuckler D. Who’s afraid of noncommunicable diseases? Raising awareness of the effects of noncommunicable diseases on global health. J Health Commun. 2011 8;16(sup2) Suppl 2:82–93. 10.1080/10810730.2011.602178 21916716

[13] Shiffman J, Smith S. Generation of political priority for global health initiatives: a framework and case study of maternal mortality. Lancet. 2007 10 13;370(9595):1370–9. 10.1016/S0140-6736(07)61579-7 17933652

[14] Frenk J, Gómez-Dantés O. False and real, but avoidable, dichotomies - Authors’ reply. Lancet. 2017 8 12;390(10095):648. 10.1016/S0140-6736(17)31443-5 28816134

